# Statistical learning leads to persistent memory: Evidence for one-year consolidation

**DOI:** 10.1038/s41598-017-00807-3

**Published:** 2017-04-10

**Authors:** Andrea Kóbor, Karolina Janacsek, Ádám Takács, Dezso Nemeth

**Affiliations:** 1 Brain Imaging Centre, Research Centre for Natural Sciences, Hungarian Academy of Sciences, Magyar tudósok körútja 2., H–1117 Budapest, Hungary; 2 Institute of Psychology, Eötvös Loránd University, Izabella utca 46., H–1064 Budapest, Hungary; 3 MTA-ELTE NAP B Brain, Memory and Language Research Group, Institute of Cognitive Neuroscience and Psychology, Research Centre for Natural Sciences, Hungarian Academy of Sciences, Magyar tudósok körútja 2., H–1117 Budapest, Hungary

## Abstract

Statistical learning is a robust mechanism of the brain that enables the extraction of environmental patterns, which is crucial in perceptual and cognitive domains. However, the dynamical change of processes underlying long-term statistical memory formation has not been tested in an appropriately controlled design. Here we show that a memory trace acquired by statistical learning is resistant to inference as well as to forgetting after one year. Participants performed a statistical learning task and were retested one year later without further practice. The acquired statistical knowledge was resistant to interference, since after one year, participants showed similar memory performance on the previously practiced statistical structure after being tested with a new statistical structure. These results could be key to understand the stability of long-term statistical knowledge.

## Introduction

Statistical learning is a fundamental mechanism of the brain which extracts and represents regularities of our environment. It is crucial in perception^1–5^, associative learning^6^, predictive processing^3, 6, 7^, and acquisition of perceptual, motor, cognitive, and social skills; thus, statistical learning underlies many day-to-day activities during the entire lifespan^8–11^. Moreover, statistical learning could be considered as the basis of language acquisition^11, 12^. Despite the extensive research on this field, the strong implicit assumption that statistical learning leads to persistent memory has not yet been empirically tested in a carefully controlled experimental design, and the dynamics of those mechanisms underlying consolidation have remained unclear. Here we show direct evidence for the one-year retention and resistance to interference of a memory trace that was acquired by statistical learning in humans.

An important challenge of neuroscience is to unravel how plasticity leads to memory formation, what are the temporal dynamics of memory formation, and how long-term memory traces are retained. Learning-related plastic changes in the brain take place not only during sessions of practice, in the so-called “online” periods, but also between sessions of practice, during the so-called “offline” periods^13^. Offline processing of learnt information is referred to as consolidation, which pertains to the stabilization of memory traces after their initial acquisition^14–20^.

Although some previous studies investigated the long-term retention of different perceptual-motor skills in humans using various tasks^21–24^, only the study of Romano *et al*.^25^ examined the long-term stability of *statistical learning*, but even these findings were limited in validity. Although the retention of statistical memory was found after one year in a small sample of perceptual-motor skill experts and older non-experts, the authors did not investigate whether statistical memory was resistant to new, interfering information. In fact, the general effect of interference on statistical learning has not been investigated within an extended time period. In this way, only the *retention* of memory traces could have been measured rather than their *consolidation*, which could not unravel the core processes underlying long-term memory formation. Therefore, the aim of the current study is twofold as follows. First, we explore the nature of those dynamic processes that underpin the long-term consolidation of statistical regularities by introducing interfering sequences in the course of learning. Second, we provide a valid replication of the study conducted by Romano *et al*.^25^ on a larger, homogeneous sample and show clear empirical evidence that statistical learning leads to persistent and immutable memory traces that are resistant to forgetting over a longer stretch of time. This combined approach enables to examine the adaptive nature of learning processes and the robustness of representations related to statistical regularities, since the change in performance measured on the previously practiced and the new, interfering sequence could be quantified.

In the current study, healthy young adults performed a statistical learning task and they were retested one year later without further practice between the two tests. Statistical learning was induced by a perceptual-motor four-choice reaction time task that, unknown to the participant, included a temporal/serial regularity between non-adjacent trials. To test the susceptibility of the acquired statistical knowledge to interference, during the testing phases, before and after the one-year delay, we changed the underlying statistical structure of the task for short periods. Thus, we administered the task with both the previously practiced statistical regularity and a new regularity that partially overlapped with the former one. This design enabled us to test not only the retention of the acquired statistical knowledge but more importantly, the resistance to interference before and after the one-year delay period.

## Material and Methods

### Participants

Forty-six healthy young adults participated in the three-session-long study and we collected data from all of them at each session. However, in the main text, retention of the acquired statistical knowledge (i.e., statistical memory) over the one-year period was only assessed for those participants who exhibited significant statistical memory before the one-year delay (see also ref. 26). By restricting the sample to these participants, we exclude the possibility of learning the statistical regularities only after the one-year delay. Twenty-nine of the 46 participants met this criterion; therefore, in the main text, one-year retention was tested in the *final sample of 29 adults* (mean age = 19.93 years, *SD* = 1.98 years; mean years of education = 13.36, *SD* = 1.72 years; 28 females). The criterion for showing significant statistical memory is specified in the Statistical Analysis section. In order to consider a possible sample selection bias that could have influenced our findings, we also present the results of the full sample (46 participants) in the Analysis of the full sample section of the Supplementary Material.

All participants in the final sample as well as in the full sample had normal or corrected-to-normal vision and none of them reported a history of any neurological and/or psychiatric condition. All participants provided written informed consent before enrollment and received course credits for taking part in the experiment. The study was approved by the United Ethical Review Committee for Research in Psychology (EPKEB) in Hungary (Approval number: 30/2012) and by the research ethics committee of Eötvös Loránd University, Budapest, Hungary. The study was conducted in accordance with the Declaration of Helsinki.

### Task

**Figure 1:**
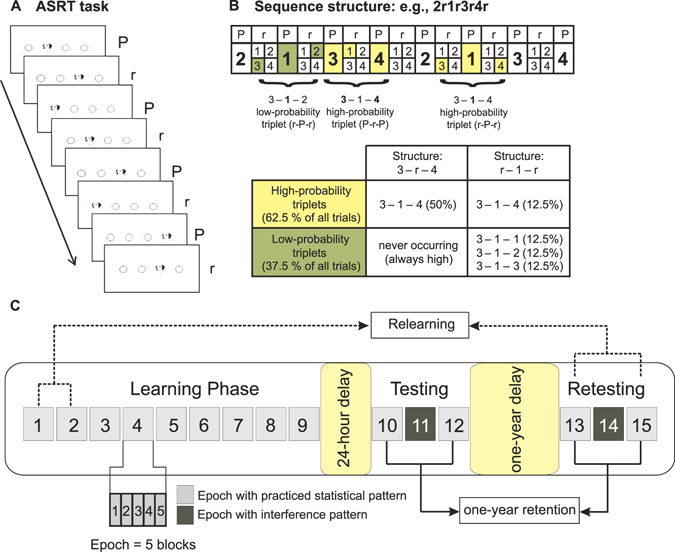
Design of the experiment. (**A**) In the Alternating Serial Reaction Time (ASRT) task, a stimulus appeared in one of four horizontally arranged empty circles on the screen. The presentation of stimuli followed an eight-element sequence, within which predetermined (P) and random (r) elements alternated with each other. (**B**) The alternating sequence in the ASRT task makes some runs of three consecutive elements (triplets) more frequent than others. High-probability triplets are denoted with yellow coloring and low-probability triplets are denoted with green coloring. (**C**) The ASRT task was administered in three sessions. During the Learning Phase, the ASRT task included nine epochs (one epoch was a cluster of five blocks, and each block consisted of 85 stimuli). Both the Testing and the Retesting Phase included only three epochs with identical structure. The middle epoch of both of these sessions (Epoch 11 and 14) served as interference with the repeating sequence being different from the one appearing in all other epochs.

The Alternating Serial Reaction Time (ASRT) task was used to induce statistical learning^18^. In this task, a stimulus (a dog’s head) appeared in one of four horizontally arranged empty circles on the screen^27^. Participants were instructed to press a corresponding key (Z, C, B, or M on a QWERTY keyboard) as quickly and accurately as possible when the stimulus occurred. Unbeknownst to the participants, the presentation of stimuli followed an eight-element sequence, within which predetermined (P) and random (r) elements alternated with each other (e.g., 2 − *r* − 1 − *r* − 3 − *r* − 4 − *r*; where numbers denote the four locations on the screen from left to right, and *r’*s denote randomly chosen locations out of the four possible ones; see Fig. 1A). There were 24 permutations of the four possible spatial positions. However, because of the continuous presentation of the stimuli, for each participant, one of the six *unique* permutations of the four possible ASRT sequence variations was selected in a pseudorandom manner^28, 29^. For details, please see the Description of sequences section of the Supplementary Material.

The alternating sequence in the ASRT task makes some runs of three consecutive elements (henceforth referred to as triplets) more frequent than others. In the example above, 2X1, 1X3, 3X4, and 4X2 (X indicates the middle element of the triplet) occurred often since the third elements could have either been a predefined or a random element (see Fig. 1B). At the same time, 1X2 and 4X3 occurred less frequently since the third element could have only been random. The former triplet types were labeled as “high-probability” triplets while the latter types were labeled as “low-probability” triplets^30^. The third element of a high-probability triplet was more predictable from the first element of the triplet than in the case of low-probability triplets. For instance, in the example shown on Fig. 1B, Position 3 as the first element of a triplet is more likely (62.5%) to be followed by Position 4 as the third element, than either Position 1, 2, or 3 (12.5%, each). In accordance with this principle, *each item* was categorized as either the third element of a high- or a low-probability triplet, and the accuracy and reaction time (RT) of the response to this item were compared between the two categories.

This task allows us to separate pure statistical learning from general skill improvements. Statistical learning is defined as faster and more accurate responses to high conditional probability events compared to that to low conditional probability ones (Fig. 1B)^18^. In contrast, general skill improvements refer to the speed-up and changes in accuracy, which are independent of the conditional probabilities of the events. These improvements reflect more efficient visuomotor and motor-motor coordination due to practice^10^.

In our study, participants were unaware of the underlying conditional probability structure of the stimulus sequence, and they did not even know that they were in a learning situation. Thus an implicit, non-conscious form of learning was tested^31, 32^. This has also been confirmed using a short questionnaire and the Inclusion-Exclusion Task (see the Testing the implicitness of the acquired statistical knowledge section).

### Procedure

One block of the ASRT task contained 85 trials (stimuli). In each block, the eight-element sequence repeated 10 times after five warm-up trials consisting only of random stimuli. The ASRT task was administered in three sessions. During the Learning Phase, the task included nine epochs, each containing five blocks (45 blocks in total). Both the Testing and the Retesting Phase included only three epochs (i.e., a total of 15 blocks of stimuli in each session), and these sessions had identical structure (Fig. 1C).

The middle epoch (5 blocks) of both of these sessions (Epoch 11 and 14) served as interference. An interference sequence was defined as a previously unpracticed repeating sequence that was different from the one appearing in all other epochs. For instance, if the original sequence was 2 − *r* − 1 − *r* − 3 − *r* − 4, the interference sequence could be 2 − *r* − 1 − *r* − 4 − *r* − 3. Thus, there was partial overlap between the original sequence and the interference sequence. Twenty-five percent of the originally high-probability triplets remained high-probability in the interference sequence, while the remaining 75% became low-probability. This means that of the 16 originally high-probability triplets, four remained unchanged (“high-high” triplets: HH) and 12 high-probability triplets became low-probability ones in the interference sequence (“high-low” triplets: HL). Of the 48 low-probability triplets, 12 became high-probability ones in the interference sequence (“low-high” triplets: LH; replacing the 12 originally high-probability, HL, triplets) and the remaining 36 were low-probability ones in both sequences (“low-low” triplets: LL). Examples and frequency statistics for each triplet type are provided in the Description of sequences section of the Supplementary Material.

Participants were not told about the change in the underlying sequence during interference blocks. In addition, they were unaware of the fact that they were going to practice the same task with the same interfering sequence one year later.

### Statistical Analyses

We followed the data analysis protocol established in previous studies using the ASRT task^18, 27, 33^ and collapsed the blocks of the task into epochs of five blocks. Therefore, the Learning Phase consisted of nine epochs, while the Testing and Retesting Phases consisted of three epochs. Epochs are labeled consecutively (1, 2, …, 15) in the remainder of paper (see Fig. 1C). Mean accuracy (ratio of correct responses) and median RT only for correct responses were determined for each participant and epoch, separately for high- and low-probability triplets. Learning scores in the Learning Phase and memory scores in the Testing and Retesting Phases were then calculated as the difference between triplet types in RT (RT for low-probability triplets minus RT for high-probability triplets) and accuracy (accuracy for high-probability triplets minus accuracy for low-probability triplets). Greater score in both measures indicates larger statistical learning/memory. To evaluate statistical learning and retention of the acquired statistical knowledge, we conducted repeated measures analyses of variance (ANOVAs) and paired-samples *t*-tests. Greenhouse-Geisser epsilon (ε) correction was used when necessary. Original *df* values and corrected *p* values (if applicable) are reported together with partial eta-squared (η_*p*_^2^) as the measure of effect size. Analyses and results concerning accuracy are only reported in the Analysis of accuracy data section of the Supplementary Material; here we focus on RT measures.

Only those participants were included in the final sample who showed significant statistical memory in the *Testing Phase*. This was evaluated *blockwise* in the following way. (1) We considered only those 10 blocks of the Testing Phase in which we presented the same repeating sequence to participants as in the Learning Phase. These blocks are henceforth referred to as *non*-*interference* blocks or epochs (the cluster of five blocks; Fig. 1C). (2) In the Testing Phase, median RT for correct responses was calculated for each participant, *block*, and triplet type. (3) Then we calculated the statistical memory score (difference in RTs for low- vs. high-probability triplets) for each participant and each block. This yielded 10 memory scores per participant. (4) A one-sample *t*-test against zero was run on these scores for each participant separately to confirm whether a participant showed any significant statistical memory. (5) If the mean of the 10 blocks deviated significantly from zero (in the positive direction), the given participant was included in the final sample. A deviation from zero was regarded as significant if the *p*-value was less than 0.050. Twenty-nine participants met this criterion (mean score = 18.68 ms, *SD* = 7.96 ms).

As the focus of the current study is on the retention of statistical memory, we performed Bayesian paired-samples *t*-tests and calculated the Bayes Factor (BF) for the relevant comparisons (see the Results section below). The BF is a statistical technique that helps conclude whether the collected data favors the null-hypothesis (*H*_0_) or the alternative hypothesis (*H*_1_); thus, the BF could be considered as a weight of evidence provided by the data^34^. It is an effective mathematical approach in consolidation studies where it is expected that the acquired evidence supports *H*_0_ rather than *H*_1_^35–37^. In this case, *H*_0_ is the lack of difference between the two means, and *H*_1_ states that the two means of memory scores differ. BFs were calculated using the JASP (version 0.8, see refs 38 and 39). Here we report BF_01_ values. According to Wagenmakers *et al*.^34^, BF_01_ values between 1 and 3 indicate anecdotal evidence for *H*_0_, while values between 3 and 10 indicate substantial evidence for *H*_0_. Conversely, while values between 1/3 and 1 indicate anecdotal evidence for *H*_1_, values between 1/10 and 1/3 indicate substantial evidence for *H*_1._ If the BF is below 1/10, 1/30, or 1/100, it indicates strong, very strong, or extreme evidence for *H*_1_, respectively. Values around one do not support either *H*_0_ or *H*_1_.

## Results

### The prerequisite of memory consolidation

**Figure 2:**
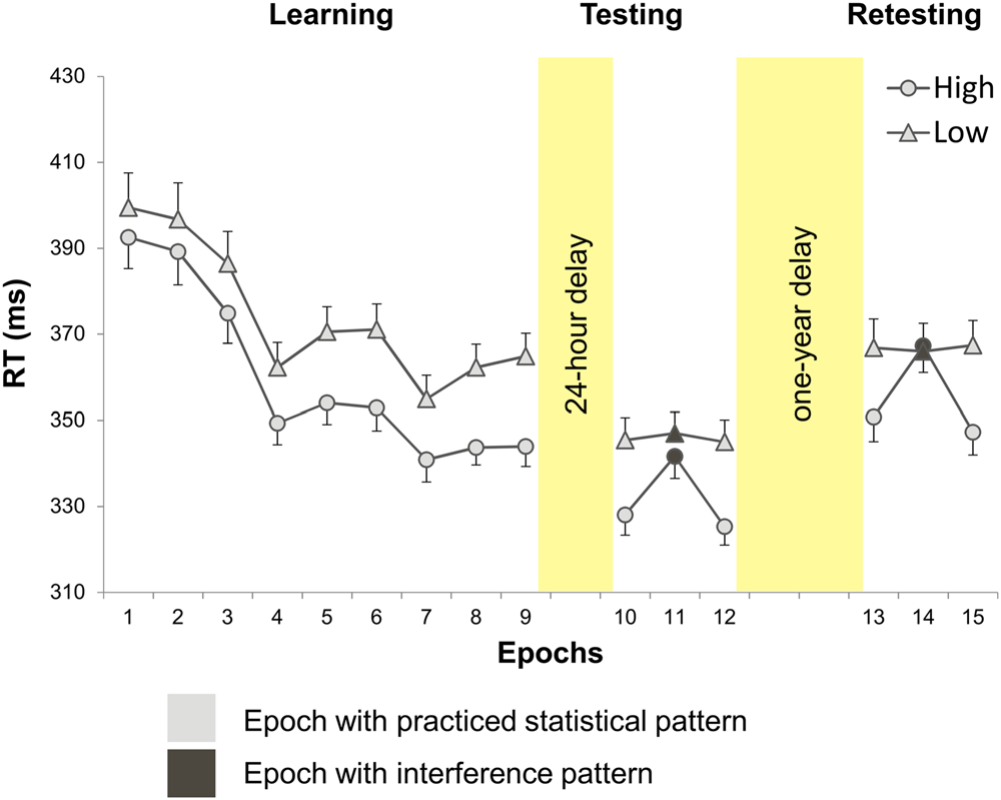
Temporal dynamics of statistical learning across epochs and sessions. Group-average (*n* = 29) RT values for correct responses as a function of the epoch (1–15) and trial type (high- vs. low-probability triplets) are presented. In the interference epochs of the Testing and Retesting Phase, LH and LL triplets corresponded to high- and low-probability triplets, respectively. Error bars denote standard error of mean.

Before memory consolidation can be assessed, significant statistical learning needs to occur preceding the long delay period, i.e., during the Learning and Testing Phases. As we report data of those participants who showed significant statistical memory in the Testing Phase, here in this analysis we only confirm that these participants – as a group – indeed exhibit significant statistical learning before the one-year delay period. Statistical learning during the *Learning Phase* was tested with a two-way repeated measures ANOVA for RT with TRIPLET (high- vs. low-probability) and EPOCH (1–9) as within-subjects factors. The ANOVA revealed significant statistical learning and general skill improvements (significant main effects of TRIPLET, *F*(1, 28) = 96.71, *p* < 0.001, η_*p*_^2^ = 0.774, and EPOCH, *F*(8, 224) = 72.08, ε = 0.303, *p* < 0.001, η_*p*_^2^ = 0.720). Participants were increasingly faster on high- than on low-probability triplets as the task progressed (TRIPLET*EPOCH interaction, *F*(8, 224) = 7.51, ε = 0.617, *p* < 0.001, η_*p*_^2^ = 0.212; see Fig. 2).

Thus, there was evidence for both statistical learning and general skill improvements during the Learning Phase. Significant statistical learning and general skill improvements before the one-year delay also took place during the *Testing Phase*, as this was the criterion for participants to be included in the final sample.

### Resistance to forgetting

**Figure 3:**
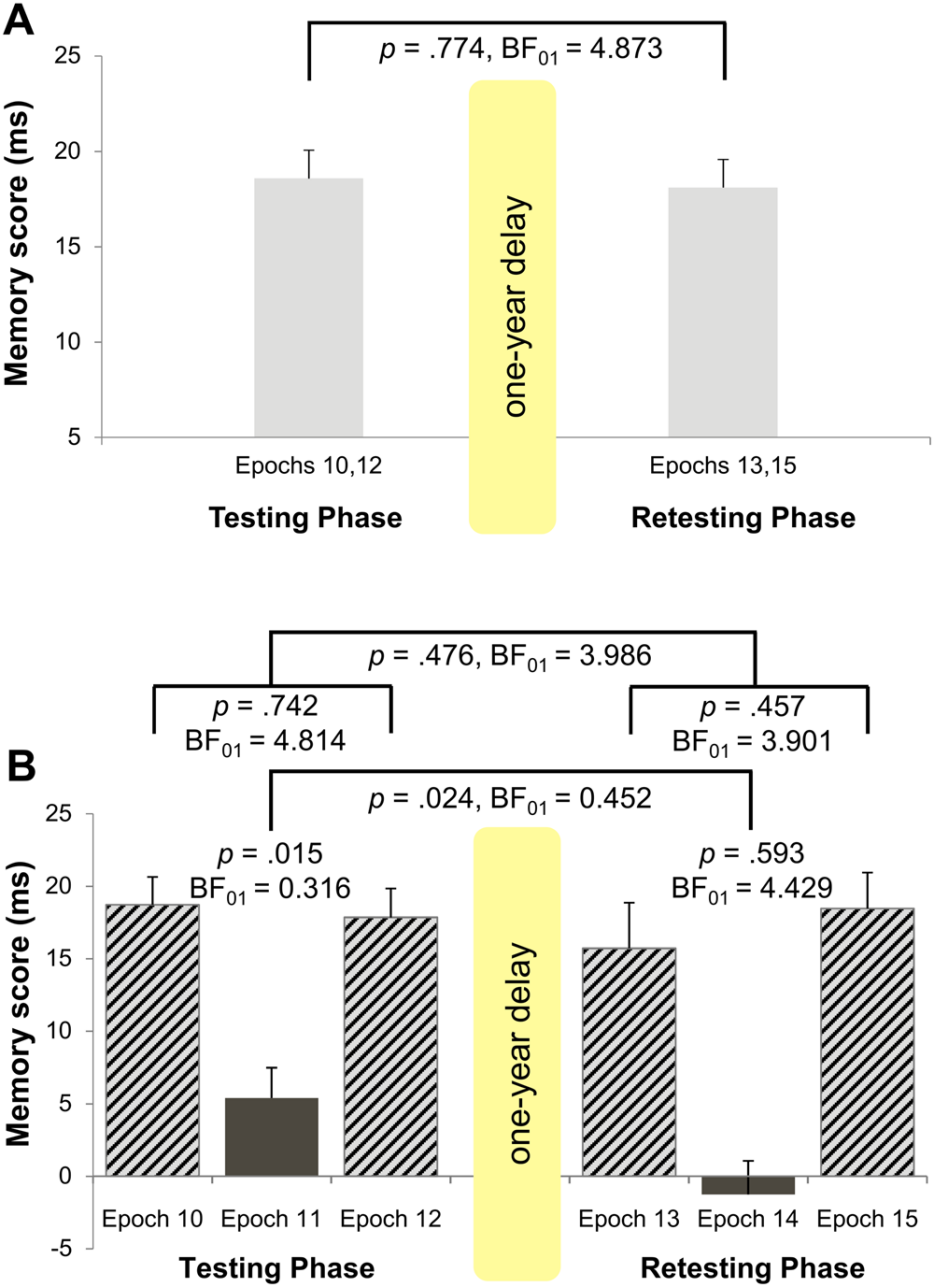
Retention of the acquired statistical knowledge. (**A**) Resistance to forgetting. Group-average (*n* = 29) of memory scores measured by RT for the non-interference epochs of the Testing (mean of Epochs 10, 12) and the Retesting Phase (mean of Epochs 13, 15). (**B**) Resistance to interference. Memory scores measured by RT for the non-interference (Epoch 10, Epoch 12, Epoch 13, Epoch 15) and interference (Epoch 11, Epoch 14) epochs of the Testing and the Retesting Phase, respectively. In subpart B, in the interference epochs, LH and LL triplets corresponded to high- and low-probability triplets, respectively. In the non-interference epochs, HL and LL triplets corresponded to high- and low-probability triplets, respectively. Error bars denote standard error of mean.

To test the one-year retention of the learned statistical contingencies, we first checked whether there is any change in statistical memory performance between the non-interference epochs of the Testing and Retesting Phases (i.e., resistance to forgetting; see Fig. 1C). An ANOVA was conducted for RT with SESSION (Testing vs. Retesting), TRIPLET (high- vs. low-probability), and EPOCH (10, 12 vs. 13, 15) as within-subjects factors. We found evidence for retained statistical memory after one-year delay (non-significant SESSION*TRIPLET interaction, *F*(1, 28) = 0.08, *p* = 0.774, η_*p*_^2^ = 0.003, BF_01_ = 4.873) with similar memory scores during Testing and Retesting Phases (see Fig. 3A and Table S1).

Irrespective of the retention of statistical memory, the same ANOVA revealed partially decreased general skills over the one-year delay. Participants were significantly slower in the Retesting Phase compared to the Testing Phase (significant main effect of SESSION: *F*(1, 28) = 24.32, *p* < 0.001, η_*p*_^2^ = 0.465, BF_01_ = 0.001; cf. Fig. 2 and Table S1). These results suggest that while statistical learning leads to persistent memory representations over the one-year delay, some aspects of general skills undergo forgetting over this period.

### The effect of the interference sequence

#### Testing Phase

The effect of interference sequence on statistical memory was evaluated in three steps. First, we tested whether significant learning of the changed statistical regularities occurred in the interference epoch. As the interference sequence was partly overlapping with the practiced sequence, some triplets in the interference epoch changed (from low- to high-probability or vice versa) and other triplets remained the same as in the non-interference epochs. To determine the extent of learning these changed probabilities, we calculated the difference in RTs and accuracy (i.e., memory scores) between those triplets that remained low-probability throughout the whole task (LL) and those ones that became high-probability in the interference epoch (LH). If participants have successfully adapted to the changed probabilities, we should find faster RTs/higher accuracy for LH triplets compared to the LL ones. Note, that the only difference between these triplets is that LH triplets became high-probability in the interference epochs, while the LL triplets did not, but otherwise both of them were low-probability in the original, practiced sequence (occurred with a similar frequency in the non-interference epochs). Again, analyses and results concerning accuracy are only reported in the Supplementary Material.

Second, if statistical memory acquired during previous practice was robust against interference, performance on the non-interference epoch (Epoch 12) *following* the interference epoch (Epoch 11) should be comparable to that on the non-interference epoch (Epoch 10) *preceding* the interference epoch. To determine whether the acquired statistical knowledge was robust against interference, we calculated the difference in RTs and accuracy (i.e., memory scores) between those triplets that remained low-probability throughout the whole task (LL) and those ones that were high-probability in the non-interference epochs but became low-probability in the interference epochs (HL). This way, we excluded those triplets from this comparison that were high-probability in the interference epoch (HH and LH). Importantly, significant statistical learning on the interference sequence was not a prerequisite of testing the resistance of the original statistical knowledge against interference.

First, we found statistical learning on the interference epoch as the statistical memory score for RT (LL minus LH) significantly differed from zero (*M* = 5.40 ms, *t*(28) = 2.58, *p* = 0.015, BF_01_ = 0.316; see Fig. 3B). Second, we compared statistical memory performance in the two non-interference epochs of the Testing Phase, and found resistance to interference since there was no significant difference between Epoch 12 and Epoch 10 (*M*_Epoch12_ = 17.86 ms vs. *M*_Epoch10_ = 18.72 ms; *t*(28) = 0.33, *p* = 0.742, BF_01_ = 4.814; see Fig. 3B). In the case of the non-interference epochs, HL and LL triplets corresponded to high- and low-probability triplets, respectively.

In summary, the significant statistical learning during interference is evidence that participants acquired new statistical information in the Testing Phase as they became faster on those high-probability triplets that has originally been low-probability ones (LH). In addition, we found efficient resistance to interference as before and after the interference, statistical memory performance remained similar on those triplets that participants rarely practiced during interference (HL).

#### Retesting Phase

To examine the effect of interference after one year has elapsed, we followed those two analysis steps described above in relation to the Testing Phase. First, we did not find statistical learning on the interference epoch as the statistical memory score for RT (LL minus LH) did not differ significantly from zero (*M* = −1.26 ms, *t*(28) = −0.54, *p* = 0.593, BF_01_ = 4.429; see Fig. 3B). Second, we compared statistical memory performance in the two non-interference epochs of the Retesting Phase, and found resistance to interference since there was no significant difference between Epoch 15 and Epoch 13 (*M*_Epoch15_ = 18.47 ms vs. *M*_Epoch13_ = 15.72 ms; *t*(28) = −0.76, *p* = 0.457, BF_01_ = 3.901; see Fig. 3B). Again, in the case of the non-interference epochs, HL and LL triplets corresponded to high- and low-probability triplets, respectively.

In summary, results suggest that participants did not acquire new statistical information on the interference sequence in the Retesting Phase as they responded with similar RTs to LH and LL triplets. At the same time, participants showed efficient resistance to interference as statistical memory performance remained similar on those triplets that participants rarely practiced during interference (HL).

#### Comparing the effect of interference sequence across Testing and Retesting Phases

First, in order to directly test whether the degree of learning new statistical information differed between the Testing and Retesting Phases, we compared the statistical memory scores between the interference epochs of the two sessions. The statistical memory score was significantly higher after 24 hours than after one year (5.40 ms vs. −1.26 ms, respectively, *t*(28) = 2.39, *p* = 0.024, BF_01_ = 0.452; see Fig. 3B).

Second, we examined whether the acquired statistical knowledge was resistant to interference across the Testing and Retesting Phases in a similar level. Particularly, we tested whether the difference in statistical memory scores between Epoch 12 and 10 and the difference in statistical memory scores between Epoch 15 and 13 were similar. Again, we compared performance on HL and LL triplets. We found evidence for the same *level of resistance to interference* in the Testing and Retesting Phases (*M*_Epoch12-10_ = −0.86 ms vs. *M*_Epoch15-13_ = 2.74 ms; *t*(28) = −0.72, *p* = 0.476, BF_01_ = 3.986; see Fig. 3B).

In sum, results suggest that statistical learning was weaker on the interference epoch in the Retesting Phase than in the Testing Phase. However, statistical knowledge was resistant to interference in a similar degree in the Testing and Retesting Phases.

### Testing relearning the statistical regularities after one-year delay

To rule out the possibility that the one-year retention of statistical memory is due to relearning in the Retesting Phase, additional ANOVAs were run with SESSION (Learning vs. Retesting Phase), TRIPLET (high- vs. low-probability), and EPOCH (1 and 2 vs. 13 and 15) as within-subjects factors (see Fig. 1C and Table S1). The significant SESSION*TRIPLET interaction (*F*(1, 28) = 25.34, *p* < 0.001, η_*p*_^2^ = 0.475) showed larger statistical memory after the one-year delay than at the beginning of the Learning Phase (*M*_Epochs13,15_ = 18.10 ms vs. *M*_Epochs1,2_ = 7.26, BF_01_ = 0.001). In sum, the learning measure confirms that participants did not relearn the task after the one-year delay, which provides further evidence for the one-year retention of statistical memory.

### Testing the implicitness of the acquired statistical knowledge

To test whether participants gained explicit/conscious knowledge about the statistical regularities underlying the ASRT task, which could have influenced both learning and consolidation processes, first we administered a short questionnaire^18^ at the end of the Retesting Phase. This questionnaire included increasingly specific questions such as “Have you noticed anything special regarding the task? Have you noticed some regularity in the sequence of stimuli”? The experimenter rated subjects’ answers on a 5-item scale, where 1 was “Nothing noticed” and 5 was “Total awareness”. None of the participants reported noticing any regularities in the task.

In addition, we administered the well-established “Process Dissociation Procedure”, PDP^40^, using an Inclusion-Exclusion task^41–45^. First, we asked the participants to generate a sequence of key presses that follows the regularity of the ASRT task, using the same response buttons as the ones they used in the ASRT task. There were four runs of the task with this *inclusion* instruction, and each run was finished after 24 key presses were made. Second, participants were asked to generate new sequences of key presses by excluding all information they (might have) consciously gained about the regularity of the ASRT task and trying to press the response buttons according to a new regularity they had never practiced before. There were four runs of the task with this *exclusion* instruction, and each run was finished after 24 key presses were made.

According to the PDP, successful performance in the inclusion condition can be achieved by using solely one’s implicit knowledge, since participants are asked to do the exact same thing as they did during performing the main task that contained the statistical regularities. Explicit knowledge in this case can further improve performance but it is not necessary for being able to perform the task successfully. Thus, in the inclusion condition, both implicit/non-conscious and explicit/conscious knowledge can lead to successful performance.

In contrast, in the exclusion condition, implicit and explicit knowledge work in opposition since only the conscious knowledge of statistical regularities can make it possible for participants to generate a different sequence of key presses and hence perform the exclusion task successfully. Generation of (thus, failure to exclude) the learned statistical regularities in the exclusion task indicates reliance on one’s implicit knowledge, which cannot be controlled consciously.

To test whether participants gained conscious knowledge of the statistical regularities, we calculated the percentage of producing high-probability triplets in the inclusion and exclusion conditions separately. Then we compared these percentages to chance level, which is 25% in this case, because out of the 64 possible triplets that participants can generate, only 16 triplets are more predictable in any of the ASRT sequences. If participants generated more high-probability triplets in the inclusion task than it would have been expected by chance, it can indicate either implicit or explicit knowledge about the statistical regularities. In contrast, if participants generated more high-probability triplets in the exclusion task than it would have been expected by chance, it can only indicate implicit knowledge and lack of conscious control over their knowledge because they *failed to exclude* this knowledge.

Participants generated 7.76% more high-probability triplets in the inclusion task than it would have been expected by chance (*t*(28) = 5.37, *p* < 0.001, BF_01_ = 4.789 × 10^−4^). More interestingly, they also generated more high-probability triplets in the exclusion task than it would have been expected by chance (6.19%; *t*(28) = 4.28, *p* < 0.001, BF_01_ = 0.007), showing that they lacked the conscious control and were unable to exclude the acquired statistical knowledge to successfully perform this part of the task. The above chance percentage of high-probability triplets did not differ between the inclusion and exclusion conditions (*t*(28) = 0.78, *p* = 0.442, BF_01_ = 3.834), suggesting that participants were relying on their implicit knowledge about the statistical regularities both in the inclusion and exclusion conditions.

In summary, these results confirm that participants did not gain explicit knowledge about the statistical regularities but used their implicit knowledge to perform the Inclusion-Exclusion Task.

## Discussion

In this study, we have shown clear evidence for the long-term consolidation of statistical memory in a carefully controlled experimental design, which involved interference manipulation. Moreover, we have highlighted how learning processes underlying statistical memory changed over a longer stretch of time. Statistical memory scores were similar after 24 hours and one year, irrespective of the type of learning measure (i.e., accuracy or RT, see Supplementary Material). Participants successfully acquired and stabilized the previously learned material, and after 24 hours, they learnt new, interfering statistical information. Statistical memory performance on the primarily practiced sequence was resistant to the interfering effect of adding a new sequence after 24 hours and one year in a comparable degree, which indicates that the acquired statistical knowledge remained persistent over time^19^.

Previous studies have shown that some aspects of skill acquisition are based on probabilistic perception and probabilistic learning^1, 11, 46, 47^. However, it has not been proven that statistical learning alone can lead to long-lasting representations, because in other studies and observations, several confounding factors were present: For instance, practice after the initial acquisition of statistical regularities together with the intervention of higher-order cognitive processes (as a result of the person intending to learn the given skill) could lead to reactivation, reconsolidation, and substantial alteration of the original memory traces. Therefore, our study took five possible confounds of consolidation into account. First, we controlled for short-term (i.e., 24-hour) consolidation of the acquired knowledge of conditional probabilities by inserting a Testing Phase. Second, we used identical design in the Testing and the Retesting Phase by inserting an interference epoch in both sessions in order to test both resistance to forgetting and resistance to interference after one year. In addition, learning on the interference sequence and resistance to interference were directly tested by quantifying how successfully participants have adapted to the *changed* probabilities in the interference and non-interference epochs. Third, we ruled out the possibility of relearning by showing better performance after the one-year period than at the very beginning of the Learning Phase. Fourth, there was no intervening practice during the one-year period, minimizing the possibility of reactivation of the acquired statistical memory during this time window. Fifth, learning was implicit because participants were unaware of the learning situation, the statistical structure of the stimulus stream, as well as of the fact that they will be tested one year later, controlling for any confounding effects of explicit strategy use during memory encoding and consolidation. Moreover, our results are supported by Bayesian statistics besides general linear models (cf. Materials and methods). Although our conclusions are based on a restricted sample of participants with robust statistical memory before the one-year delay, results of the full sample also indicate that the acquired statistical memory trace was resistant to interference as well as to forgetting after one year (see the Analysis of the full sample section of the Supplementary Material). With the latter analyses, we could ensure that the results are not influenced by a possible sample selection bias. Therefore, in regard to the applied rigorous methodology, results of the present study further extend the findings of Romano *et al*.^25^ about the long-term retention of statistical regularities.

The retention of statistical knowledge after the long delay extends the findings of Nemeth and Janacsek^14^ and Meier and Cock^16^, who found comparable retention of sequential memory across 12-hour, 24-hour, and one-week delay intervals. It is conceivable that those processes related specifically to the *retention* of statistical knowledge do not change already after 12-hour delay (see also refs 29 and 48), which is also in line with our finding that the acquired statistical knowledge was equally robust to interference both after 24 hours and one year. Our results are also comparable to the findings of Arciuli and Simpson^49^ showing stable, consistent, and sleep-independent statistical learning over the medium term (30 min, 1-, 2-, 4-, and 24-hour) in a different statistical learning task (embedded triplet paradigm).

In our design, general skill improvements refer to general speed-up, independent of the statistical structure of the task, reflecting more general learning mechanisms. Previous studies^14, 16^ found improved general skills both after 24 hours and one week compared to the end of the training session, but the degree of improvement did not differ between the two delay intervals. Moreover, retained general skills were also found after one year^25^. In the present study, general skills were retained over the one-year period measured by accuracy (see Resistance to forgetting section of the Supplementary Material) but were decreased measured by RT (i.e., slower overall RT). It is possible that the lack of practice on the ASRT task might have affected only the speed and not the precision of visuomotor coordination, which resulted in slower RTs after the one-year delay. This finding suggests that some aspects of general skills undergo forgetting over one year if no further practice is intervened. However, overall accuracy and RT were decreased after one year as compared to the *beginning* of the first session (see the main effects of SESSION in Table S1), suggesting that the general skill was retained at least in some degree (cf. ref. 25). The latter evidence also corroborates our previous statement about the implausibility of relearning the ASRT task after the offline period. Nevertheless, future studies need to disentangle how these aspects of general skills consolidate over a longer stretch of time (cf. ref. 21). In the neuroscience of skill learning, a long-standing issue is that general skill learning mechanisms are heavily intertwined with statistical or sequence-specific learning, which hinders the possibility to draw conclusions about statistical learning itself. Following the protocol of previous studies^50, 51^, here we separated pure statistical learning from these mechanisms and directly investigated the one-year retention of pure statistical learning.

Our results suggesting that the representation of the original statistical structure is immune to interference and learning a similar but new statistical structure is more demanding extend the study of Gebhart *et al*.^52^. In an auditory statistical learning task where two different statistical structures (artificial languages) determined the presented stimuli, Gebhart *et al*.^52^ showed that participants could learn only the first structure of speech streams if no explicit information was given about the change in structure during the task or the second structure was not presented for a longer duration. Accordingly, it is conceivable that more blocks of the interference sequence in our design could have increased the chance to relearn the interference sequence after one year elapsed, and the primacy effect (see also e.g., refs 53–56) of the first statistical pattern could be disrupted. We should note that the difference in learning scores in the interference epochs between 24 hours and one year was relatively small in the restricted sample, which might have changed if more chance to practice had been provided. In the full sample, the acquisition of new statistical information on the interference sequence after 24 hours was limited, and no difference was found in the learning scores of the interference epochs between 24 hours and one year. Therefore, we could not show robust evidence for the change in learning a new statistical structure over a long delay. Importantly, as the Gebhart *et al*.^52^ study showed, learning a new structure did not attenuate performance on the original structure, which was also the case in our study after the 24-hour delay (i.e., resistance to interference). Nevertheless, it still remains unclear whether far more practice on the interference sequence could cause performance deterioration on the non-interference sequence, or the different representations of the two statistical structures could be maintained at the same time and individuals could flexibly switch between them.

An advantage of having a more stable or elaborated primary structure is that the underlying cognitive/perceptual mechanisms remain sensitive to this structure later, even if the organism has to learn other statistical regularities in the meantime^52^. Meanwhile, it has been shown that stable initial memory representations disabled learning transfer between different memory tasks^57^. The long-term impact of the primarily acquired statistical structure and its predictive power have also been demonstrated in the perception of the auditory environment^58, 59^ and in processing native and non-native phonetic features of word stress^60^ as indicated by event-related brain potentials (e.g., the mismatch negativity). In line with the previous studies, our results suggest the more influential role of the primarily learnt statistical structure.

Results of the present study are also compatible with the strong impression coming from daily experience that skills, such as speaking a language or playing tennis, once acquired, are persistent throughout life. Moreover, by giving insight to the dynamic change of underlying learning processes, we could provide an experimentally well-controlled design and a possible explanatory framework for other studies investigating the long-term retention of statistical structures embedded in other perceptual/cognitive domains under more natural circumstances. For instance, on a small sample of participants, Frank *et al*.^12^ found retention of large-scale artificial languages even after three years, although participants were only exposed to these languages for 10 days without directly paying attention to the presented chunks of languages. The authors claimed this was evidence that statistical learning skills related to speech segmentation could be applied to the lexicons of natural languages. A simple paradigm such as the ASRT task might be used over an even longer time period to test the upper bound of the retention of statistical knowledge, and to obtain a clearer insight to the characteristics of processes determining consolidation in such a large-scale as language acquisition (see also ref. 61).

Taken together, the present study shows that probabilistic mechanisms are not only present in perception and learning but also that their results remain stable over longer periods of time. Specifically, we demonstrated that statistical knowledge was resistant to interference and also to forgetting after one year. Our experimental design also enabled to test how the neurocognitive processes underlying statistical learning changed over this time period. In the long run, these results can help build a better computational framework^46^ of systems-level brain mechanisms that underlie learning and memory.

## Electronic supplementary material

Supplementary Material

## References

[1] Orbán G, Fiser J, Aslin RN, Lengyel M (2008). Bayesian learning of visual chunks by human observers. Proc Natl Acad Sci.

[2] Fiser J, Aslin RN (2002). Statistical learning of new visual feature combinations by infants. Proc Natl Acad Sci.

[3] Winkler I, Denham SL, Nelken I (2009). Modeling the auditory scene: predictive regularity representations and perceptual objects. Trends Cogn Sci.

[4] Yang Z, Purves D (2003). A statistical explanation of visual space. Nat Neurosci.

[5] Teinonen T, Fellman V, Näätänen R, Alku P, Huotilainen M (2009). Statistical language learning in neonates revealed by event-related brain potentials. BMC Neurosci.

[6] Turk-Browne NB, Scholl BJ, Johnson MK, Chun MM (2010). Implicit perceptual anticipation triggered by statistical learning. J Neurosci.

[7] Bar M (2007). The proactive brain: using analogies and associations to generate predictions. Trends Cogn Sci.

[8] Kaufman SB (2010). Implicit learning as an ability. Cognition.

[9] Ullman MT (2004). Contributions of memory circuits to language: the declarative/procedural model. Cognition.

[10] Hallgato E, Gyori-Dani D, Pekar J, Janacsek K, Nemeth D (2013). The differential consolidation of perceptual and motor learning in skill acquisition. Cortex.

[11] Saffran JR, Aslin RN, Newport EL (1996). Statistical Learning by 8-Month-Old Infants. Science.

[12] Frank MC, Tenenbaum JB, Gibson E (2013). Learning and Long-Term Retention of Large-Scale Artificial Languages. PLoS One.

[13] Genzel L, Robertson EM (2015). To Replay, Perchance to Consolidate. PLoS Biol.

[14] Nemeth D, Janacsek K (2011). The dynamics of implicit skill consolidation in young and elderly adults. J Gerontol B Psychol Sci Soc Sci.

[15] Robertson EM (2009). From Creation to Consolidation: A Novel Framework for Memory Processing. PLoS Biol.

[16] Meier B, Cock J (2014). Offline consolidation in implicit sequence learning. Cortex.

[17] Krakauer JW, Shadmehr R (2006). Consolidation of motor memory. Trends Neurosci.

[18] Song S, Howard JH, Howard DV (2007). Sleep does not benefit probabilistic motor sequence learning. J Neurosci.

[19] Robertson EM, Pascual-Leone A, Miall RC (2004). Current concepts in procedural consolidation. Nat Rev Neurosci.

[20] Robertson EM (2012). New Insights in Human Memory Interference and Consolidation. Curr Biol.

[21] Hikosaka O (2002). Long-term retention of motor skill in macaque monkeys and humans. Exp Brain Res.

[22] Willingham DB, Dumas JA (1997). Long-term retention of a motor skill: Implicit sequence knowledge is not retained after a one-year delay. Psychol Res.

[23] Ammons RB (1958). Long-term retention of perceptual-motor skills. J Exp Psychol.

[24] Fleishman EA, Parker JF (1962). Factors in the retention and relearning of perceptual-motor skill. J Exp Psychol.

[25] Romano JC, Howard JH, Howard DV (2010). One-year retention of general and sequence-specific skills in a probabilistic, serial reaction time task. Memory.

[26] Albouy G (2008). Both the hippocampus and striatum are involved in consolidation of motor sequence memory. Neuron.

[27] Nemeth D, Janacsek K, Polner B, Kovacs ZA (2013). Boosting human learning by hypnosis. Cereb Cortex.

[28] Howard JH, Howard DV (1997). Age differences in implicit learning of higher order dependencies in serial patterns. Psychol Aging.

[29] Nemeth D (2010). Sleep has no critical role in implicit motor sequence learning in young and old adults. Exp Brain Res.

[30] Nemeth D, Janacsek K, Fiser J (2013). Age-dependent and coordinated shift in performance between implicit and explicit skill learning. Front Comput Neurosci.

[31] Reber AS (1989). Implicit learning and tacit knowledge. J Exp Psychol Gen.

[32] Cleeremans, A. & Dienes, Z. In *The Cambridge Handbook of Computational Modeling* (ed. R. Sun) 396–421 (Cambridge University Press, 2008).

[33] Virag M (2015). Competition between frontal lobe functions and implicit sequence learning: evidence from the long-term effects of alcohol. Exp Brain Res.

[34] Wagenmakers EJ, Wetzels R, Borsboom D, van der Maas HL (2011). Why psychologists must change the way they analyze their data: the case of psi: comment on Bem (2011). J Pers Soc Psychol.

[35] Wagenmakers EJ (2007). A practical solution to the pervasive problems of p values. Psychon Bull Rev.

[36] Dienes, Z. Using Bayes to get the most out of non-significant results. *Front Psychol* **5**, 10.3389/fpsyg.2014.00781 (2014). 10.3389/fpsyg.2014.00781 PMC4114196 25120503

[37] Dienes Z (2011). Bayesian Versus Orthodox Statistics: Which Side Are You On?. Perspect Psychol Sci.

[38] JASP Team JASP (Version 0.8.0.0) [Computer software]. URL https://jasp-stats.org/ (2016).

[39] Rouder JN, Speckman PL, Sun D, Morey RD, Iverson G (2009). Bayesian t tests for accepting and rejecting the null hypothesis. Psychon Bull Rev.

[40] Jacoby LL (1991). A process dissociation framework: Separating automatic from intentional uses of memory. J Mem Lang.

[41] Destrebecqz A, Cleeremans A (2001). Can sequence learning be implicit? New evidence with the process dissociation procedure. Psychon Bull Rev.

[42] Destrebecqz A (2005). The neural correlates of implicit and explicit sequence learning: Interacting networks revealed by the process dissociation procedure. Learn Memory.

[43] Jimenez L, Vaquero JM, Lupianez J (2006). Qualitative differences between implicit and explicit sequence learning. J Exp Psychol Learn Mem Cogn.

[44] Fu Q, Dienes Z, Fu X (2010). Can unconscious knowledge allow control in sequence learning?. Conscious Cogn.

[45] Fu Q, Dienes Z, Fu X (2010). The distinction between intuition and guessing in the SRT task generation: a reply to Norman and Price. Conscious Cogn.

[46] Fiser J, Berkes P, Orban G, Lengyel M (2010). Statistically optimal perception and learning: from behavior to neural representations. Trends Cogn Sci.

[47] Fiser J, Aslin RN (2001). Unsupervised statistical learning of higher-order spatial structures from visual scenes. Psychol Sci.

[48] Press DZ, Casement MD, Pascual-Leone A, Robertson EM (2005). The time course of off-line motor sequence learning. Cognitive Brain Res.

[49] Arciuli J, Simpson IC (2012). Statistical learning is lasting and consistent over time. Neurosci Lett.

[50] Hunt RH, Aslin RN (2001). Statistical learning in a serial reaction time task: access to separable statistical cues by individual learners. J Exp Psychol Gen.

[51] Howard DV (2004). Implicit sequence learning: effects of level of structure, adult age, and extended practice. Psychol Aging.

[52] Gebhart AL, Aslin RN, Newport EL (2009). Changing Structures in Midstream: Learning Along the Statistical Garden Path. Cogn Sci.

[53] Junge JA, Scholl BJ, Chun MM (2007). How is spatial context learning integrated over signal versus noise? A primacy effect in contextual cueing. Vis Cogn.

[54] da Estrela C, Byers-Heinlein K (2015). Vois-Tu Le Kem? Do You See the Bos? Foreign Word Learning at 14 Months. Infancy.

[55] Yu RQ, Zhao J (2015). The persistence of the attentional bias to regularities in a changing environment. Atten Percept Psychophys.

[56] Billig AJ, Carlyon RP (2016). Automaticity and primacy of auditory streaming: Concurrent subjective and objective measures. J Exp Psychol Human.

[57] Mosha N, Robertson EM (2016). Unstable Memories Create a High-Level Representation that Enables Learning Transfer. Curr Biol.

[58] Mullens D (2014). Altering the primacy bias—How does a prior task affect mismatch negativity?. Psychophysiology.

[59] Todd J, Provost A, Cooper G (2011). Lasting first impressions: A conservative bias in automatic filters of the acoustic environment. Neuropsychologia.

[60] Honbolygó F, Csépe V (2013). Saliency or template? ERP evidence for long-term representation of word stress. Int J Psychophysiol.

[61] Morgan-Short K, Finger I, Grey S, Ullman MT (2012). Second Language Processing Shows Increased Native-Like Neural Responses after Months of No Exposure. PLoS One.

