# Supplementary material for: Statistical learning leads to persistent memory: Evidence for one-year consolidation
Source: Sci Rep. 2017 Apr 10;7:760. doi: 10.1038/s41598-017-00807-3 (PMC5429700; doi:10.1038/s41598-017-00807-3)
Supplement: Supplementary file 1 — Supplementary Material [file 41598_2017_807_MOESM1_ESM.doc]

**Supplementary Material of the manuscript entitled**

**Statistical learning leads to persistent memory: Evidence for one-year consolidation**

**Authors: Andrea Kóbor, Karolina Janacsek, Ádám Takács, Dezso Nemeth**

**Analysis of accuracy data**

In the ASRT task, accuracy of responding is influenced by the feedback given to participants after each block [^1^](#_ENREF_1). Namely, participants received feedback about their overall RT and accuracy in the given block, and they were encouraged to keep accuracy around 92%. In addition, since overall accuracy has usually been high with relatively low variability in samples of healthy young adults performing the ASRT task [^2-4^](#_ENREF_2) (the overall mean accuracy in this study is 94.9%, *SD* = 2.4%, *n* = 29), RT could be regarded as a more appropriate measure of statistical learning, which explains why we reported RT data in the main text. Analyses of accuracy data yielded similar results and conclusions to that of the RT. Here we report the analyses of accuracy restricted to those participants who exhibited significant statistical memory before the one-year delay (*n* = 29).

***Learning Phase***

Statistical learning during the Learning Phase was tested with a two-way repeated measures ANOVA for accuracy with TRIPLET (high- vs. low-probability) and EPOCH (1–9) as within-subjects factors. The ANOVA revealed significant statistical learning (significant main effect of TRIPLET, *F*(1, 28) = 56.22, *p* < .001, η*_p_*^2^ = .668) and general skill improvements (significant main effect of EPOCH, *F*(8, 224) = 11.24, ε = .607, *p* < .001, η*_p_*^2^ = .286). Participants became less accurate on low-probability triplets than on high-probability ones as the task progressed (significant interaction of TRIPLET*EPOCH, *F*(8, 224) = 2.99, ε = .739, *p* = .009, η*_p_*^2^ = .096; see Fig. S1).

***Resistance to forgetting***

An ANOVA was conducted for accuracy with SESSION (Testing vs. Retesting), TRIPLET (high- vs. low-probability), and EPOCH (10, 12 vs. 13, 15) as within-subjects factors. We found evidence for retained statistical memory after one-year delay (non-significant SESSION*TRIPLET interaction, *F*(1, 28) < 0.01, *p* = .995, η*_p_*^2^ < .001, BF_01_ = 5.066) with similar memory scores during Testing and Retesting Phases (see Fig. S2A and Table S1). In regard to general skills, participants showed similar level of accuracy in the Testing and Retesting Phases (non-significant main effect of SESSION, *F*(1, 28) = 0.05, *p* = .818, η*_p_*^2^ = .002, BF_01_ = 4.937; cf. Table S1).

***The effect of the interference sequence: Testing Phase***

First, we found statistical learning on the interference epoch as the statistical memory score (calculated as the difference between accuracy for those triplets that became high-probability, LH, and those that remained low-probability, LL) significantly differed from zero (*M* = 2.27%, *t*(28) = 2.24, *p* = .033, BF_01_ = 0.593; see Fig. S2B). Second, we compared statistical memory performance in the two non-interference epochs of the Testing Phase (calculated as the difference between accuracy for those triplets that were high-probability in the non-interference epochs but became low-probability in the interference epoch, HL, and those that remained low-probability, LL) and found resistance to interference since there was no significant difference between Epoch 12 and Epoch 10 (*M*_Epoch12_ = 2.4% vs. *M*_Epoch10_ = 3.8%; *t*(28) = 1.70, *p* = .101, BF_01_ = 1.419; see Fig. S2B).

***The effect of the interference sequence: Retesting Phase***

First, we did not find statistical learning on the interference epoch as the statistical memory score did not differ significantly from zero (*M* = 1.4%, *t*(28) = 1.59, *p* = .124, BF_01_ = 1.657; see Fig. S2B). Second, we found resistance to interference since there was no significant difference between Epoch 15 and Epoch 13 (*M*_Epoch15_ = 3.5% vs. *M*_Epoch13_ = 2.8%; *t*(28) = -0.70, *p* = .491, BF_01_ = 4.052; see Fig. S2B).

***Comparing the effect of interference sequence across Testing and Retesting Phases***

Statistical memory scores in the Testing and Retesting Phases did not differ significantly, *t*(28) = 0.72, *p* = .475, BF_01_ = 3.982; see Fig. S2B. We found evidence for the same level of resistance to interference in the Testing and Retesting Phases (*M*_Epoch12-10_ = -1.4% vs. *M*_Epoch15-13_ = 0.7%; *t*(28) = -1.65, *p* = .110, BF_01_ = 1.513; see Fig. S2B).

***Testing relearning the statistical regularities after one-year delay***

The significant SESSION*TRIPLET interaction (*F*(1, 28) = 14.27, *p* = .001, η*_p_*^2^ = .338) showed larger statistical memory after the one-year delay than at the beginning of the Learning Phase (*M*_Epochs13,15_ = 3.8% vs. *M*_Epochs1,2_ = 1.3%, BF_01_ = 0.024).

**Supplementary Figures**

**
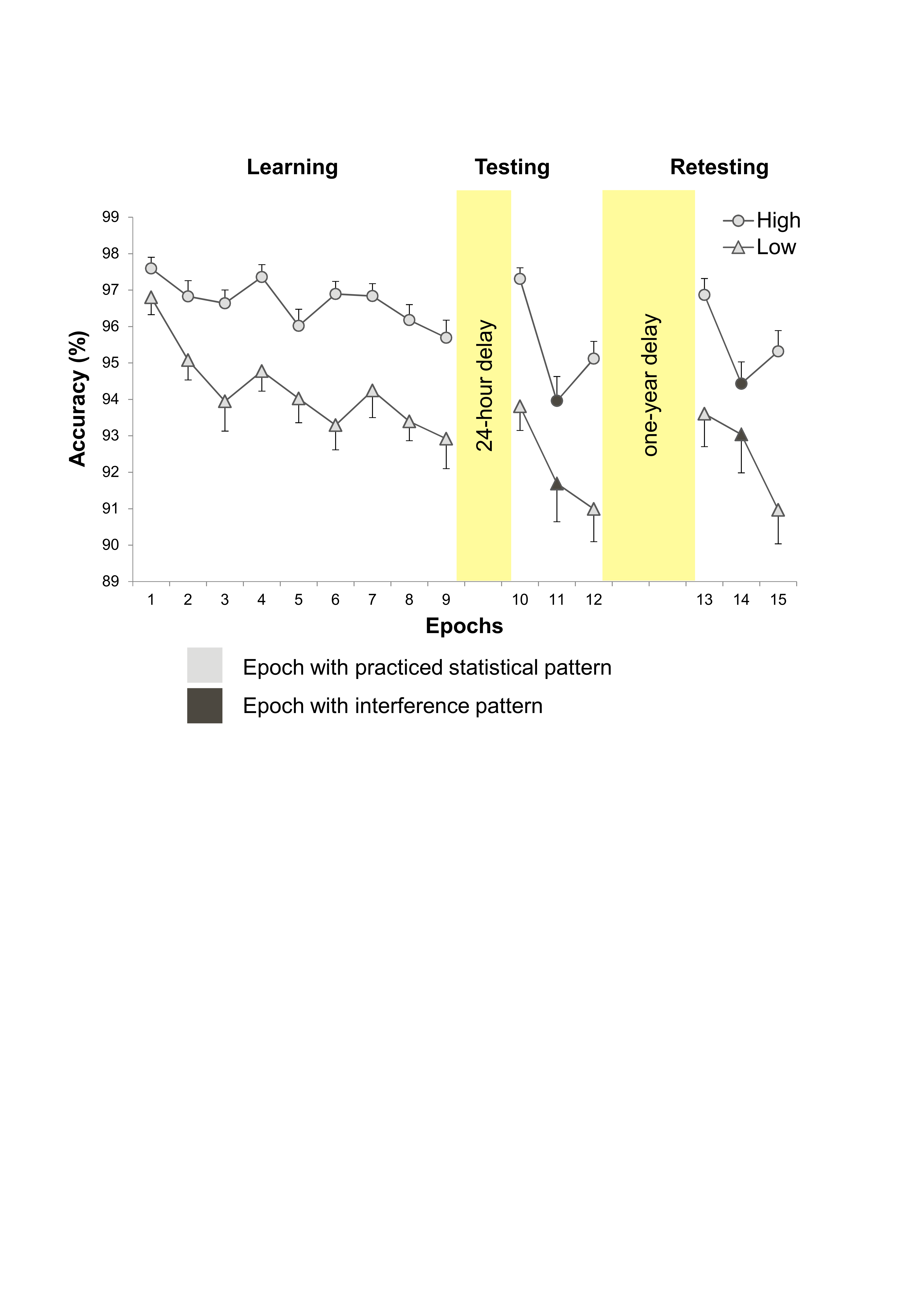
**

Fig. S1. Group-average (*n* = 29) accuracy values as a function of the epoch (1–15) and trial type (high- vs. low-probability triplets) are presented. In the interference epochs of the Testing and Retesting Phase, LH and LL triplets corresponded to high- and low-probability triplets, respectively. Error bars denote standard error of mean.


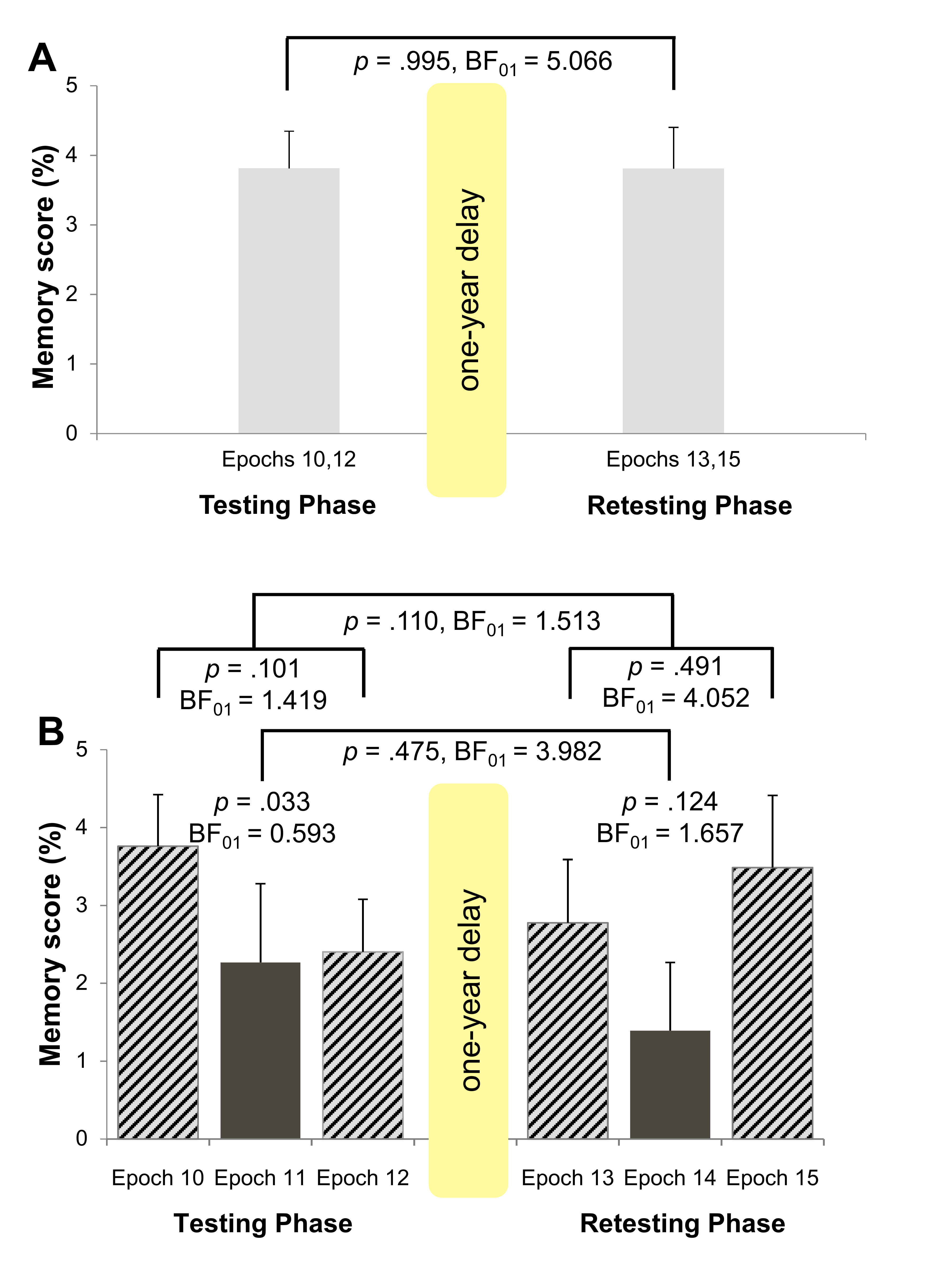


Fig. S2. Retention of the acquired statistical knowledge. (A) Resistance to forgetting. Group-average (*n* = 29) of memory scores measured by accuracy for the non-interference epochs of the Testing (mean of Epochs 10, 12) and the Retesting Phase (mean of Epochs 13, 15). (B) Resistance to interference. Memory scores measured by RT for the non-interference (Epoch 10, Epoch 12, Epoch 13, Epoch 15) and interference (Epoch 11, Epoch 14) epochs of the Testing and the Retesting Phase, respectively. In subpart B, in the interference epochs, LH and LL triplets corresponded to high- and low-probability triplets, respectively. In the non-interference epochs, HL and LL triplets corresponded to high- and low-probability triplets, respectively. Error bars denote standard error of mean.

**Supplementary Table**

Table S1. Summary of results from ANOVAs performed on accuracy and RT measures in relation to the one-year retention (*n* = 29).

|  | SESSION | | | TRIPLET | | | EPOCH | | | SESSION*TRIPLET | | | SESSION*EPOCH | | | TRIPLET*EPOCH | | | SESSION*TRIPLET*EPOCH | | |
| --- | --- | --- | --- | --- | --- | --- | --- | --- | --- | --- | --- | --- | --- | --- | --- | --- | --- | --- | --- | --- | --- |
| Accuracy | *F* | *p* | η*_p_*^2^ | *F* | *p* | η*_p_*^2^ | *F* | *p* | η*_p_*^2^ | *F* | *p* | η*_p_*^2^ | *F* | *p* | η*_p_*^2^ | *F* | *p* | η*_p_*^2^ | *F* | *p* | η*_p_*^2^ |
| One-yr retention | 0.05 | .818 | .002 | 64.65 | **<.001** | .698 | 46.78 | **<.001** | .626 | <0.01 | .995 | .000 | 0.80 | .379 | .028 | 3.22 | .083 | .103 | 0.23 | .639 | .008 |
| Relearning | 26.00 | **<.001** | .481 | 58.83 | **<.001** | .678 | 42.56 | **<.001** | .603 | 14.27 | **.001** | .338 | 2.72 | .111 | .088 | 6.45 | **.017** | .187 | 0.01 | .907 | .000 |
| RT | *F* | *p* | η*_p_*^2^ | *F* | *p* | η*_p_*^2^ | *F* | *p* | η*_p_*^2^ | *F* | *p* | η*_p_*^2^ | *F* | *p* | η*_p_*^2^ | *F* | *p* | η*_p_*^2^ | *F* | *p* | η*_p_*^2^ |
| One-yr retention | 24.32 | **<.001** | .465 | 225.80 | **<.001** | .890 | 0.78 | .385 | .027 | 0.08 | .774 | .003 | <0.01 | .954 | .000 | 3.73 | .064 | .118 | 0.30 | .589 | .011 |
| Relearning | 37.90 | **<.001** | .575 | 115.75 | **<.001** | .805 | 1.81 | .190 | .061 | 25.34 | **<.001** | .475 | 0.19 | .666 | .007 | 1.28 | .267 | .044 | 0.46 | .502 | .016 |

*Note.* In order to directly evaluate one-year retention, three-way repeated measures ANOVAs were conducted for accuracy and for RTs with SESSION (Testing vs. Retesting Phase), TRIPLET (high- vs. low-probability), and EPOCH (10 and 12 vs. 13 and 15) as within-subjects factors. The interaction of SESSION*TRIPLET and/or SESSION*TRIPLET*EPOCH indicate whether statistical memory has been influenced by the session in which the task was administered; namely, whether performance has changed after one year. Similar ANOVAs were conducted to rule out the possibility of relearning in the Retesting Phase. In this case, the first two epochs of the Learning Phase were compared to Epoch 13 and 15 of the Retesting Phase. SESSION*TRIPLET and/or SESSION*TRIPLET*EPOCH interactions indicate whether the statistical regularities were only relearned in the Retesting Phase. *p*-values below .050 are **boldfaced**.

**Analyis of the full sample**

In order to take into consideration a possible sample selection bias that could have influenced our results, we performed the main analyses on the full sample (*N* = 46), as well. The full sample also included those participants who did not show significant statistical memory before the one-year delay. Analysis of the full sample yielded similar results and conclusions to that of the restricted sample in regard to RT and accuracy: The acquired statistical memory trace was resistant to interference as well as to forgetting after one year.

***Learning Phase***

Statistical learning during the Learning Phase was tested with a two-way repeated measures ANOVA with TRIPLET (high- vs. low-probability) and EPOCH (1–9) as within-subjects factors for RT and accuracy, respectively. The ANOVA revealed significant statistical learning (significant main effect of TRIPLET, *RT*: *F*(1, 45) = 140.54, *p* < .001, η*_p_*^2^ = .757; *accuracy*: *F*(1, 45) = 102.46, *p* < .001, η*_p_*^2^ = .695) and general skill improvements (significant main effect of EPOCH, *RT*: *F*(8, 360) = 113.06, ε = .388, *p* < .001, η*_p_*^2^ = .715; *accuracy*: *F*(8, 360) = 18.84, ε = .692, *p* < .001, η*_p_*^2^ = .295). Participants were increasingly faster on high- than on low-probability triplets and less accurate on low-probability triplets than on high-probability ones as the task progressed (significant interaction of TRIPLET*EPOCH, *RT*: *F*(8, 360) = 11.50, ε = .714, *p* < .001, η*_p_*^2^ = .203, *accuracy*: *F*(8, 360) = 3.39, ε = .784, *p* = .003, η*_p_*^2^ = .070). Thus, there was evidence for both statistical learning and general skill improvements during the Learning Phase in the full sample.

***Resistance to forgetting***

An ANOVA was conducted with SESSION (Testing vs. Retesting), TRIPLET (high- vs. low-probability), and EPOCH (10, 12 vs. 13, 15) as within-subjects factors for RT and accuracy, respectively. We found evidence for retained statistical memory after one-year delay (non-significant SESSION*TRIPLET interaction, *RT*: *F*(1, 45) = 0.72, *p* = .400, η*_p_*^2^ = .016; *M*_Epochs10,12_ = 14.93 ms vs. *M*_Epochs13,15_ = 16.00 ms, BF_01_ = 4.454; *accuracy*: *F*(1, 45) = 0.15, *p* = .700, η*_p_*^2^ = .003; *M*_Epochs10,12_ = 3.6% vs. *M*_Epochs13,15_ = 3.5%, BF_01_ = 5.824) with similar memory scores during Testing and Retesting Phases. In regard to general skills, participants showed slower RT in the Retesting Phase than in the Testing Phase but similar level of accuracy (main effect of SESSION, *RT*: *F*(1, 45) = 42.10, *p* < .001, η*_p_*^2^ = .483; *M*_Epochs10,12_ = 338.81 ms vs. *M*_Epochs13,15_ = 363.40 ms, BF_01_ = 4.195 x 10^-6^; *accuracy*: *F*(1, 45) = 1.28, *p* = .264, η*_p_*^2^ = .028; *M*_Epochs10,12_ = 93.8% vs. *M*_Epochs13,15_ = 94.3%, BF_01_ = 3.433). Thus, while statistical memory was persistent over the one-year delay, some aspects of general skills underwent forgetting in the full sample.

***The effect of the interference sequence: Testing Phase***

First, in terms of *RT*, we did not find statistical learning on the interference epoch as the statistical memory score (calculated as the difference between RT for those triplets that remained low-probability, LL, and those that became high-probability, LH) did not differ significantly from zero (*M* = 1.74 ms, *t*(45) = 0.85, *p* = .401, BF_01_ = 4.460). In terms of *accuracy*, we found statistical learning on the interference epoch as the statistical memory score significantly differed from zero (*M* = 1.64%, *t*(45) = 2.39, *p* = .021, BF_01_ = 0.489).

Second, we compared statistical memory performance in the two non-interference epochs of the Testing Phase (calculated as the difference between RT for those triplets that that remained low-probability, LL, and those that were high-probability in the non-interference epochs but became low-probability in the interference epoch, HL) and found resistance to interference since there was no significant difference between Epoch 12 and Epoch 10 both in terms of *RT* (*M*_Epoch12_ = 13.51 ms vs. *M*_Epoch10_ = 14.67 ms; *t*(45) = 0.63, *p* = .535, BF_01_ = 5.198) and *accuracy* (*M*_Epoch12_ = 3.3% vs. *M*_Epoch10_ = 3.6%; *t*(45) = 0.32, *p* = .754, BF_01_ = 5.964). In sum, regarding the full sample, the acquisition of new statistical information on the interference sequence in the Testing Phase was limited, as participants responded with similar RTs but different accuracy to LH and LL triplets. At the same time, we found that the previously learnt statistical memory was resistant to interference.

***The effect of the interference sequence: Retesting Phase***

First, we did not find statistical learning on the interference epoch as the statistical memory score did not differ significantly from zero either for *RT* (*M* = -0.63 ms, *t*(45) = -0.34, *p* = .738, BF_01_ = 5.925) or *accuracy* (*M* = 0.18%, *t*(45) = 0.24, *p* = .810, BF_01_ = 6.081). Second, we found resistance to interference since there was no significant difference between Epoch 15 and Epoch 13 both in terms of *RT* (*M*_Epoch15_ = 15.89 ms vs. *M*_Epoch13_ = 14.10 ms; *t*(45) = -0.67, *p* = .508, BF_01_ = 5.069) and *accuracy* (*M*_Epoch15_ = 3.8% vs. *M*_Epoch13_ = 2.7%; *t*(45) = -1.33, *p* = .191, BF_01_ = 2.752). Regarding the full sample, results suggest that participants did not acquire new statistical information on the interference sequence in the Retesting Phase; however, we found efficient resistance to interference.

***Comparing the effect of interference sequence across Testing and Retesting Phases***

In regard to the interference sequence, statistical memory scores in the Testing and Retesting Phases did not differ significantly either for *RT* (*t*(45) = 0.98, *p* = .331, BF_01_ = 3.976) or for *accuracy* (*t*(28) = 1.57, *p* = .124, BF_01_ = 2.012). We found evidence for the same level of resistance to interference in the Testing and Retesting Phases both for *RT* (*M*_Epoch12-10_ = -1.16 ms vs. *M*_Epoch15-13_ = 1.97 ms; *t*(28) = -0.83, *p* = .410, BF_01_ = 4.514) and *accuracy* (*M*_Epoch12-10_ = -0.3% vs. *M*_Epoch15-13_ = 1.1%; *t*(28) = -1.19, *p* = .240, BF_01_ = 3.227). Results of the full sample suggest that the lack of statistical learning on the interference epoch was similar between Testing and Retesting Phases, and the original statistical knowledge was resistant to interference in a similar degree in the Testing and Retesting Phases.

***Testing relearning the statistical regularities after one-year delay***

The significant SESSION*TRIPLET interaction (*RT*: *F*(1, 45) = 28.64, *p* < .001, η*_p_*^2^ = .389; *accuracy*: *F*(1, 45) = 15.32, *p* < .001, η*_p_*^2^ = .254) showed larger statistical memory after the one-year delay than at the beginning of the Learning Phase (*RT*: *M*_Epochs13,15_ = 16.0 ms vs. *M*_Epochs1,2_ = 6.56 ms, BF_01_ = 1.588 x 10^-4^; *accuracy*: *M*_Epochs13,15_ = 3.5% vs. *M*_Epochs1,2_ = 1.3%, BF_01_ = 0.012). These results confirm that participants did not relearn the task after the one-year delay.

**Description of sequences**

***ASRT sequences***

There were 24 permutations of the four possible spatial positions. The first participant received the first permutation, the second received the second one, and so on; thus, all permutations were used in the experiment. Nevertheless, because of the continuous presentation of the stimuli, for instance, the sequences 2 – *r* – 1 – *r* – 3 – *r* – 4, 1 – *r* – 3 – *r* – 4 – *r* – 2, 3 – *r* – 4 – *r* – 2 – *r* – 1, and 4 – *r* – 2 – *r* – 1 – *r* – 3 (numbers denote the four locations on the screen from left to right, and *r’*s denote randomly chosen locations out of the four possible ones) were considered identical as they contained the same triplets. This means that there were six *unique* ASRT sequences in the experiment (2 – *r* – 1 – *r* – 3– *r* – 4, 2 – *r* – 1 – *r* – 4 – *r* – 3, 2 – *r* – 3 – *r* – 1 – *r* – 4, 2 – *r* – 3 – *r* – 4 – *r* – 1, 2 – *r* – 4 – *r* – 1 – *r* – 3, 2 – *r* – 4 – *r* – 3 – *r* – 1).

***Relation between the original and the interference sequences***

There was partial overlap between the original sequences and the interference sequences. 25% of the originally high-probability triplets remained high-probability in the interference sequence, while the remaining 75% became low-probability triplets. If the original sequence was 2 – *r* – 1 – *r* – 3 – *r* – 4, the interference sequence could be 2 – *r* – 1 – *r* – 4 – *r* – 3. This way, the 2 – X – 1 triplets (X indicates the middle element of the triplet; i.e., 2 – 1 – 1, 2 – 2 – 1, 2 – 3 – 1, 2 – 4 – 1) were high-probability ones in both the original and the interference sequence. Following this example, of the 16 originally high-probability triplets (i.e., 2 – X – 1, 1 – X – 3, 3 – X – 4, 4 – X – 2), four remained unchanged (triplets 2 – X – 1) and 12 high-probability triplets became low-probability ones in the interference sequence.

Beyond the 16 high-probability triplets, there were 48 low-probability triplets for the given sequence. Specifically, there were four possible positions for the first element of the triplet, four for the second, and only three for the third, because the fourth position corresponded to the high-probability triplets (discussed in the previous paragraph); i.e., 4 * 4 * 3 = 48. In the case of the interference sequence, of the 48 low-probability triplets, 12 became high-probability ones (in the above example: 1 – X – 4, 4 – X – 3, 3 – X – 2). The remaining 36 low-probability triplets were low-probability ones in both sequences (e.g., 2 – X – 3, 2 – X – 4, 1 –X – 2, 4 – X – 1).

In regard to the frequency statistics, as it is typical in the ASRT task because of the alternating sequence structure, 62.5% of all triplets were high-probability triplets (i.e., all pattern trials and by chance, one-fourth of random trials form high-probability triplets) and 37.5% of them were low-probability ones (three-fourth of random trials) for a given sequence. Therefore, each of the 16 high-probability triplets occurred with a frequency of 3.91% and each of the low-probability ones occurred with a frequency of 0.78%. The frequency of the different triplet types was as follows in the interference epoch: four high-probability triplets remained unchanged, corresponding to 15.64% of the trials (the so-called “high-high” triplets, HH, 4 * 3.91%), and 12 high-probability triplets became low, corresponding to 46.92% of the trials (“high-low” triplets, HL, 12 * 3.91%), adding up to 62.5% of all trials. Of the 37.5% low-probability triplets, 28.08% remained unchanged (“low-low” triplets, LL, 36 * 0.78%), while 9.36% became high-probability ones (“low-high” triplets, LH, 12 * 0.78%). Triplet types, considering the change in the sequence in the interference epoch, could be summarized as HH = 15.64%, HL = 46.92%, LL = 28.08%, LH = 9.36%. Note, however, that these percentages do not mean that LL triplets were more frequent than HH triplets because there were more individual triplets within the LL category, each appearing 0.78% of the time, in contrast to high-probability triplets, in which case each triplet occurred 3.91% of the time. Thus, irrespective of what the actual high- and low-probability triplets were in the non-interference or interference epochs, high-probability triplets individually were *always* about 5 times more frequent than low-probability triplets. The calculations in this paragraph are just to gain a better understanding of how the sequence was changed in the interference epoch compared to the non-interference epochs.

**References**

1 Romano, J. C., Howard, J. H., Jr. & Howard, D. V. One-year retention of general and sequence-specific skills in a probabilistic, serial reaction time task. *Memory* **18**, 427-441, doi:10.1080/09658211003742680 (2010).

2 Howard, J. H., Jr. & Howard, D. V. Age differences in implicit learning of higher order dependencies in serial patterns. *Psychol Aging* **12**, 634-656, doi:10.1037/0882-7974.12.4.634 (1997).

3 Nemeth, D. *et al.* Sleep has no critical role in implicit motor sequence learning in young and old adults. *Exp Brain Res* **201**, 351-358, doi:10.1007/s00221-009-2024-x (2010).

4 Nemeth, D. & Janacsek, K. The dynamics of implicit skill consolidation in young and elderly adults. *J Gerontol B Psychol Sci Soc Sci* **66**, 15-22, doi:10.1093/geronb/gbq063 (2011).
